# The ModA2 Phasevarion of nontypeable *Haemophilus influenzae* Regulates Resistance to Oxidative Stress and Killing by Human Neutrophils

**DOI:** 10.1038/s41598-017-03552-9

**Published:** 2017-06-09

**Authors:** Kenneth L. Brockman, M. Taylor Branstool, John M. Atack, Frank Robledo-Avila, Santiago Partida-Sanchez, Michael P. Jennings, Lauren O. Bakaletz

**Affiliations:** 1 Center for Microbial Pathogenesis, The Research Institute at Nationwide Children’s Hospital and The Ohio State University College of Medicine, Columbus, Ohio, 43205 USA; 2 Institute for Glycomics, Griffith University, Gold Coast, Queensland, 4222 Australia

## Abstract

Nontypeable *Haemophilus influenzae* (NTHI) is the causative agent of multiple respiratory tract infections. Several human pathogens, including NTHI, possess a novel genetic system, termed the phasevarion, which mediates a rapid and reversible change in the expression of many genes throughout the chromosome. This occurs by phase variation of a single gene (*modA*) that encodes a DNA methyltransferase and results in two phenotypically distinct subpopulations, ON and OFF. NTHI encounters many pressures within the various microenvironments of its human host as the disease course evolves from one of asymptomatic nasopharyngeal carriage to overt disease. These include oxidative stresses, which are present throughout the respiratory tract. To persist in the human nasopharynx and as a pathogen throughout the airways, NTHI must be able to mitigate toxic levels of oxidative stress. Here we show that expression of ModA2, *modA2* ON status, resulted in increased sensitivity to oxidative stress. Furthermore, the *modA2* ON status resulted in decreased resistance to neutrophil-mediated killing, which resulted in selection for the *modA2* OFF subpopulation in an *ex vivo* survival assay. These findings highlight the importance of the ModA2 phasevarion in adaptation to innate host defences and reveal an additional microenvironmental pressure that selected for a specific ModA2 subpopulation.

## Introduction

Nontypeable *Haemophilus influenzae* (NTHI) commonly resides asymptomatically in the human nasopharynx, but can also cause multiple respiratory tract diseases. NTHI is the primary causative agent of chronic and recurrent otitis media (OM)^1^ and can give rise to many additional respiratory diseases, which include acute OM, rhinosinusitis, bronchitis and exacerbations in patients with cystic fibrosis and chronic obstructive pulmonary disease, among others^2–5^. NTHI must rapidly adapt to changes in the microenvironment in order to survive and persist within the varied niches of the human respiratory tract. One strategy NTHI utilizes for adaptation is the phasevarion, or phase variable regulon ^6, 7^.

Several important mucosa-associated human pathogens have evolved to use a phasevarion to rapidly regulate a simultaneous change in the expression of multiple genes throughout the chromosome. This switch in gene expression occurs via phase variation of a DNA methyltransferase, encoded by the *mod* gene. A switch in *mod* status, from OFF to ON or *vice versa*, alters the NTHI transcriptional profile and results in two distinct bacterial subpopulations each with unique gene expression patterns and phenotypes^6^. The phasevarion mechanism, has been identified in *Haemophilus influenzae* ^8, 9^, *Streptococcus pneumoniae* ^10^ *, Moraxella catarrhalis* ^11^, *Helicobacter pylori* ^12^ and *Neisseria* species^13, 14^. The phasevarions of these human pathogens regulate the expression of multiple virulence factors and affect experimental disease severity. We have shown that the NTHI ModA2 phasevarion regulates genes involved in iron acquisition, and phenotypes such as antibiotic resistance and biofilm formation^8^. In a chinchilla model of OM, the *modA2* ON status is preferentially selected for and a shift from a *modA2* OFF population to a *modA2* ON population within the chinchilla middle ear significantly increases the severity of disease^15^. By regulation of multiple virulence factors, this unique genetic mechanism allows NTHI to rapidly adapt to changes in host microenvironments and to evade host immune defence mechanisms.

The production of reactive oxygen species is an important antimicrobial host defence that occurs within the human respiratory pathways. As such, bacteria have developed several methods to combat these oxidative stressors in order to survive within the microenvironments of the airway. NTHI utilizes several mechanisms to combat oxidative stress, which include expression of the enzymes catalase and peroxidredoxin-gluteredoxin as well as proteins that restrict free iron within the cell^16–18^. Therefore, in this study we sought to determine the effect of the ModA2 phasevarion on the resistance of NTHI to multiple sources of oxidative stress.

## Results

### Generation of NTHI strain 723 *modA2* variants unable to phase vary

This study utilized the clinical isolate NTHI strain 723 as the representative strain for the ModA2 phasevarion. The wild type clinical isolate of this strain, contains a region of tetrameric repeats within the *modA2* open reading frame. These repeats result in phase variable expression of the ModA2 methyltransferase^8^. To better investigate the contribution of each variant subtype, *modA2* ON or *modA2* OFF, to resistance against oxidative stress, we constructed NTHI strain 723 variants that were unable to phase vary. NTHI in which the repeats of *modA2* were either removed, 0rep *modA2* OFF, or reduced to a single repeat, 1rep *modA2* ON were generated. The resultant bacteria were unable to phase vary, or switch the status of *modA2*, and will be referred to as *modA2* locked OFF and *modA2* locked ON, respectively. By removing the ability to phase vary, we aimed to be able to directly assign phenotypes without the added complication of *modA2* switching during growth under stress conditions. This allowed a clear assessment of phenotypes based on *modA2* ON/OFF status. Growth under aerobic conditions confirmed that the phase variable and locked subpopulations all grew at similar rates in sBHI broth culture (data not shown). The *modA2* locked variants were utilized for all experiments presented herein.

### Resistance of *modA2* ON and *modA2* OFF to reactive oxygen species

**Figure 1:**
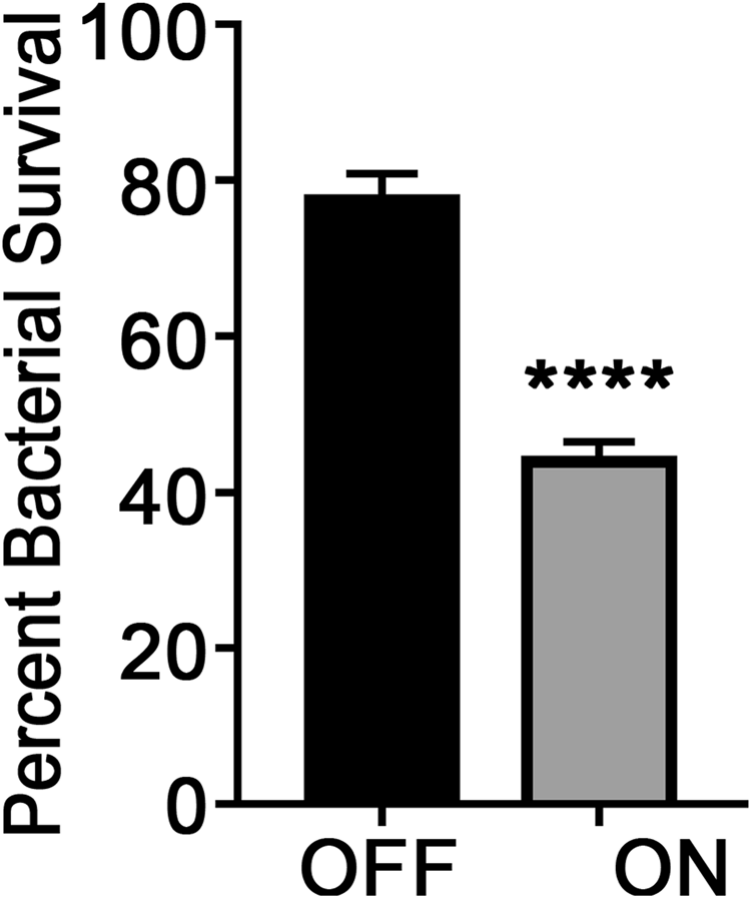
The *modA2* locked ON variant was more sensitive to killing due to H_2_O_2_ than the *modA2* locked OFF variant. *modA2* locked ON and *modA2* locked OFF were exposed to 1 mM H_2_O_2_ for 10 min and survival relative to untreated cells was determined. *modA2* locked ON exhibited significantly reduced survival in the presence of H_2_O_2_ compared to *modA2* locked OFF. *P* < 0.0001, unpaired t-test.

To determine if ModA2 expression impacted resistance to oxidative stress, we tested 723 *modA2* locked ON and locked *modA2* OFF for resistance to exogenous hydrogen peroxide. We found that *modA2* locked ON was significantly more sensitive to killing by H_2_O_2_ compared to *modA2* locked OFF (Fig. 1, *P* < 0.0001). This finding suggested that *modA2* ON was less equipped to handle high levels of oxidative stress compared to *modA2* OFF. These data further suggested that continuous or repeated exposure to H_2_O_2_ stress may result in an advantageous selection for a *modA2* OFF subpopulation.

**Figure 2:**
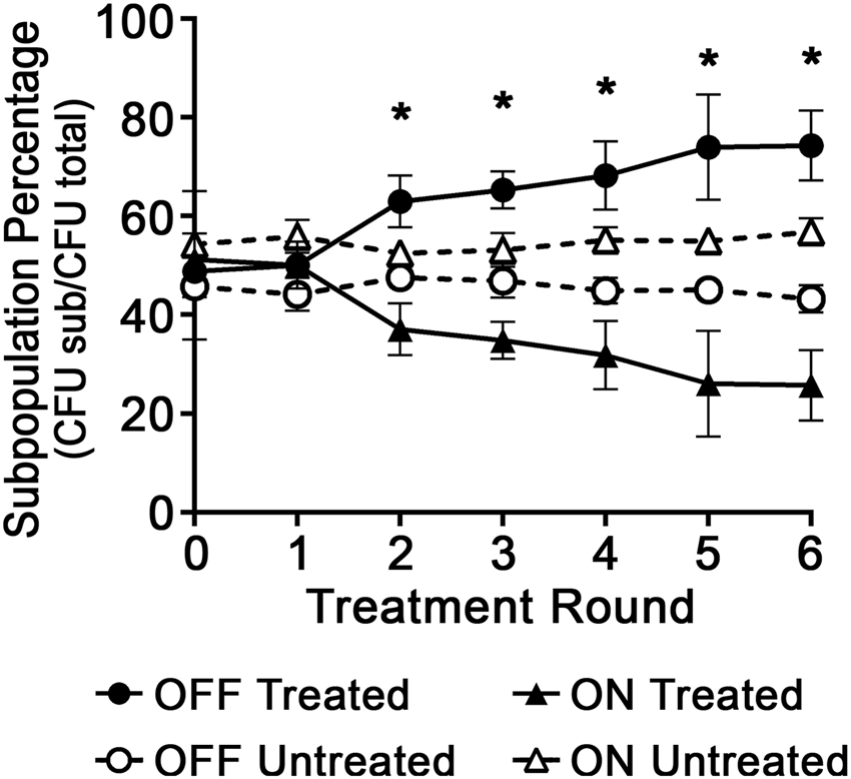
The *modA2* locked OFF subpopulation was selected for during repeated exposure to oxidative stress induced by H_2_O_2_. A mixed culture that contained an equal number of *modA2* locked ON:GFP and *modA2* locked OFF:mCherry was subjected to six rounds of H_2_O_2_ exposure. Selection for *modA2* locked OFF was apparent after 2 rounds of stress, whereas after 6 rounds, the *modA2* locked OFF subpopulation comprised 75% of the total population (solid markers). The percentages of each subpopulation were significantly different at rounds 2 to 6, *P* < 0.01, unpaired t-test. There was no significant difference between the percentages of each population at any time point for untreated populations (hollow markers), unpaired t-test. Exogenous H_2_O_2_ selected for the *modA2* OFF status over the *modA2* ON status.

To test this hypothesis, a mixed population, comprised of equal CFU of *modA2* locked ON and *modA2* locked OFF, was exposed to repeated periods of H_2_O_2_ stress. To track each subpopulation, *modA2* locked ON carried pMDC-P1, which constitutively expressed GFP, and *modA2* locked OFF carried pKM1.1, which constitutively expressed the mCherry fluorophore. In these plasmids, the promoter of the outer membrane protein OMP P2 drove the constitutive expression of mCherry and the promoter of OMP P5 drove the constitutive expression of GFP. The mixed population was exposed to 1 mM H_2_O_2_ for 10 minutes, and the individual bacterial subpopulations were enumerated by serial dilution and plate counts. The culture was then allowed to recover for 10 minutes in the absence of exogenous H_2_O_2_. A total of six rounds of H_2_O_2_ exposure were carried out and the relative abundances of the *modA2* locked ON and *modA2* locked OFF subpopulations were determined. Repeated exposure to H_2_O_2_ resulted in a selection for the *modA2* locked OFF subpopulation. A significant selection for the *modA2* locked OFF population had occurred after two rounds of stress (Fig. 2, *P* < 0.005, unpaired t-test) and further selection for the *modA2* locked OFF subpopulation continued through six rounds of treatment (Fig. 2, treated). There was no significant change in the relative abundance of either subpopulation when the mixed culture was supplemented with DPBS in the place of H_2_O_2_ (Fig. 2, untreated). To rule out a contribution of the expressed fluorophore to the results observed, a mixture of *modA2* locked ON:mCherry and *modA2* locked OFF:GFP was also tested. A similar selection for *modA2* locked OFF was observed in these fluorophore swap studies (data not shown). Based on these results, we predicted that *modA2* OFF cells may have an overall selective advantage over *modA2* ON cells under conditions of H_2_O_2_ stress *in vivo*.

### Role of genes involved in resistance to oxidative stress

**Figure 3:**
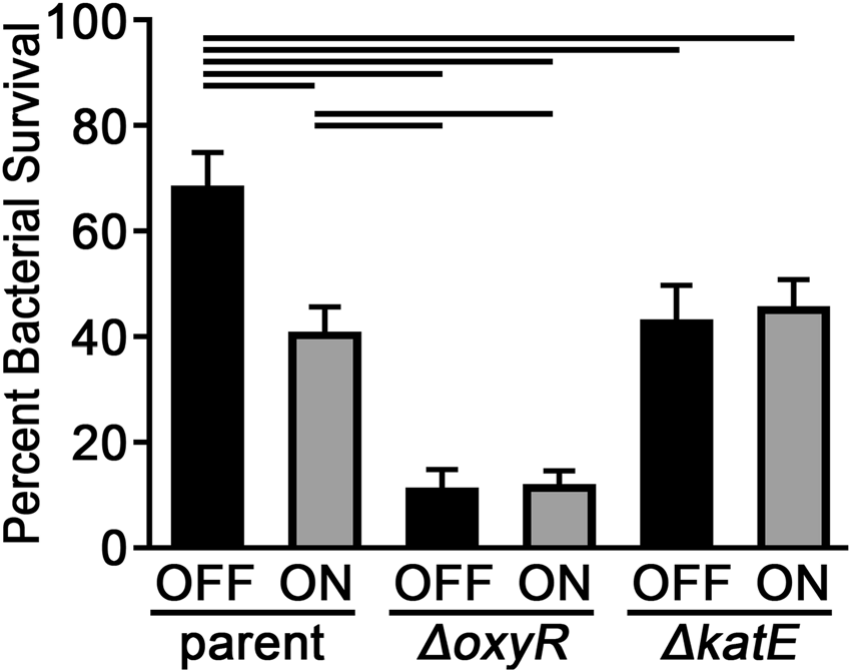
ModA2-mediated regulation of H_2_O_2_ resistance is independent of OxyR. Mutants that lacked *oxyR*, had significantly decreased survival when exposed to H_2_O_2_ stress compared to either parent. In contrast to the parents, there was no significant difference in survival between the *oxyR* mutants themselves. There was no significant difference between the survival of the Δ*katA modA2* locked ON and the Δ*katA modA2* locked OFF mutants, and the survival of both Δ*katA* mutants was similar to that of the *modA2* locked ON parent. Statistical differences were determined by 1-way ANOVA with Tukey’s multiple comparisons; lines above bars indicate significance, Alpha = 0.05.

In response to oxidative stress, OxyR regulates genes involved in oxidative stress resistance, including catalase, an enzyme that catalyses the decomposition of H_2_O_2_ into oxygen and water. To determine if OxyR played a role in the observed differences in resistance to H_2_O_2_, we constructed a pair of mutants that lacked the transcriptional regulator OxyR. NTHI strain 723 mutants that lack *oxyR* were constructed in both the *modA2* locked ON and *modA2* locked OFF backgrounds. Both mutants, Δ*oxyR modA2* locked ON and Δ*oxyR modA2* locked OFF were significantly less resistant to oxidative stress than either of the parents (Fig. 3, *P* < 0.01, 1way ANOVA with multiple comparisons). Furthermore, in contrast to what was observed with the parent variants, there was no significant difference in resistance to oxidative killing between the Δ*oxyR* mutants, regardless of *modA2* status (Fig. 3, *P* = 0.9). These data suggested that ModA2-mediated regulation of H_2_O_2_ resistance directly or indirectly required the presence of OxyR.

**Figure 4:**
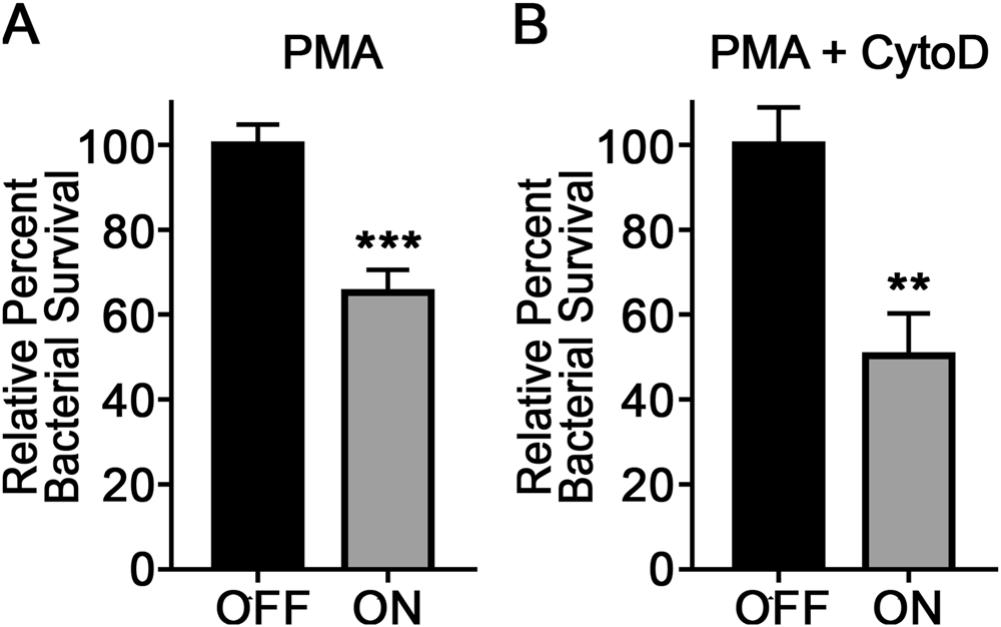
The *modA2* locked ON variant was more sensitive to killing by human neutrophils. NTHI were incubated with human neutrophils that had been activated by addition of PMA without (**A**) or with (**B**) cytochalasin D pretreatment. After 30 minutes, the relative percent survival was calculated by comparison of viable (CFU) *modA2* locked ON to *modA2* locked OFF. (**A**) *modA2* locked ON had significantly reduced survival in the presence of activated neutrophils compared to *modA2* locked OFF, ***P < 0.001, unpaired t-test. (**B**) When cytochalasin D was used to prevent phagocytosis *modA2* locked ON still had significantly reduced survival compared to *modA2* locked OFF, **P < 0.005, unpaired t-test. The *modA2* ON status provided less resistance to killing by neutrophils compared to the *modA2* OFF status.

To further validate this hypothesis, we generated a NTHI strain 723 mutant pair that lacked the gene that encodes catalase, *katA* (called *hktE* in some strains). In NTHI, catalase is regulated as part of the OxyR regulon^19^. As anticipated, the Δ*katA* mutants showed no significant difference in resistance to H_2_O_2_ when the Δ*katA modA2* locked ON and Δ*katA modA2* locked OFF mutants were compared (Fig. 3, *P* = 0.79). Interestingly, the survival of both mutants was similar to that of the *modA2* locked ON parent (Fig. 3, *P* > 0.99, 1way ANOVA, multiple comparisons). These results suggested that the *modA2* locked ON parent was inherently as susceptible to oxidative stress as a catalase mutant, yet the *modA2 locked* OFF parent was not (Fig. 4, *P* < 0.005, 1way ANOVA multiple comparisons).

### ModA2 regulated resistance to neutrophil-mediated killing

Resistance to oxidative stress is critical for the survival and pathogenesis of host-adapted pathogens. In addition to oxidative stress generated during aerobic respiration by bacteria of the respiratory tract, innate immune defenses of the host rely heavily on the release of reactive oxygen species (ROS) and other antimicrobial compounds. ROS are a major component of phagolysosomes during intracellular killing and large amounts of ROS are released from neutrophils during the oxidative burst^20, 21^. To define the role of the ModA2 phasevarion in a more host relevant *ex vivo* model of innate immune response, we assessed the relative killing of *modA2* locked ON and *modA2* locked OFF by human neutrophils.

The *modA2* locked ON and *modA2* locked OFF subpopulations were exposed to neutrophils that had been highly activated by addition of phorbol 12-myristate 13-acetate (PMA). PMA is a known agonist of protein kinase C (PKC), which when stimulated activates NADPH-oxidase and subsequent production of ROS^22^. *modA2* locked ON had significantly reduced survival in the presence of activated neutrophils compared to *modA2* locked OFF (Fig. 4A, *P* < 0.0005, unpaired t-test). Neutrophils kill bacteria through phagocytosis as well as the release of ROS, antimicrobial peptides, proteases and NETs into the microenvironment^22–24^. To determine bacterial killing specifically due to ROS released as a product of the oxidative burst, neutrophils were pretreated with cytochalsin D, an inhibitor of actin polymerization, which prevented phagocytosis. When phagocytosis was blocked, *modA2* locked ON was still significantly more sensitive to neutrophil-derived exogenous ROS compared to *modA2* locked OFF (Fig. 4B, *P* < 0.005, unpaired t-test). Pretreatment of neutrophils with diphenyleneiodonium (DPI), an antagonist of NADPH-oxidase, prevented the release of ROS, but was also directly toxic to NTHI and therefore unable to be used in this assay^23, 25, 26^. These data suggested that the increased sensitivity of *modA2* locked ON to oxidative stress lead to greater bacterial killing due to ROS generated by human neutrophils, as was seen with addition of exogenous H_2_O_2_ in the *in vitro* assays above.

**Figure 5:**
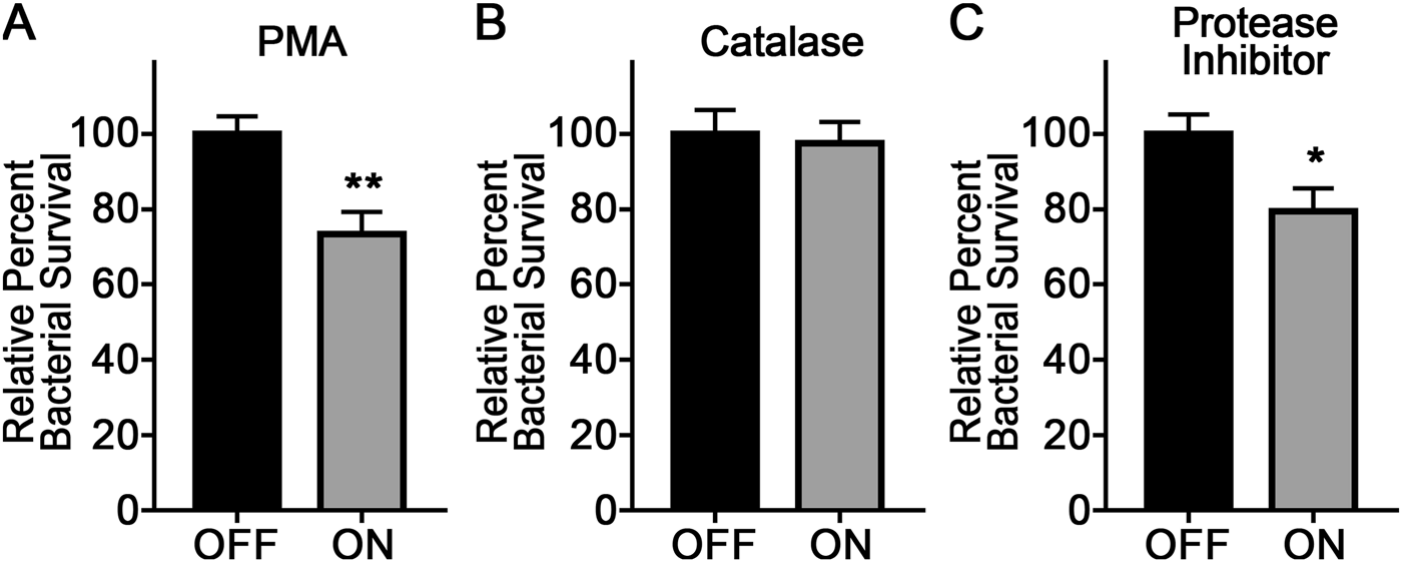
Hydrogen peroxide was the primary compound responsible for the increased neutrophil-mediated killing of *modA2* locked ON. NTHI survival was determined following exposure to antimicrobials released from activated neutrophils. (**A**) *modA2* locked ON was significantly more sensitive to supernatants of PMA activated neutrophils. (**B**) Treatment with catalase to degrade H_2_O_2_ abolished the increased killing of *modA2* locked ON. (**C**) Treatment with a protease inhibitor cocktail to block neutrophil-derived proteases, known to increase opsonophagocytic killing, did not significantly alter relative survival of *modA2* locked ON compared to *modA2* locked OFF. These results indicated that H_2_O_2_ released by neutrophils was primarily responsible for the increased killing of *modA2* locked ON.

Neutrophils release a multitude of antimicrobial agents as part of the innate immune response. To confirm which components released by neutrophils were responsible for the increased killing of *modA2* locked ON, extracellular milieu from activated neutrophils were treated with antimicrobial inhibitors and tested for the ability to kill NTHI. Human neutrophils were activated by the addition of PMA as described above, and extracellular supernatants were collected after 20 minutes at the peak concentration of free extracellular ROS (see Supplementary Figure 1). Cell free supernatants were treated with inhibitors for 5 minutes at 37 °C. NTHI *modA2* locked ON or *modA2* locked OFF were added to the supernatants and incubated for an additional 10 minutes at 37 °C. The bacteria were then collected and enumerated by serial dilution and plating to determine relative bacterial killing. Supernatants collected from PMA activated neutrophils without additional treatment resulted in relative bacterial killing similar to that observed with the addition of exogenous H_2_O_2_ (Fig. 5A, *P* < 0.01, unpaired t-test). However, no significant difference in survival was observed when bacteria were exposed to supernatants that had been treated with catalase to enzymatically breakdown free H_2_O_2_ (Fig. 5B, *P* = 0.78, unpaired t-test). Removal of free H_2_O_2_ from the medium abrogated the decreased survival of *modA2* locked ON in the presence of untreated supernatants (Fig. 5A vs. B), and confirmed the specific contribution of neutrophil-derived exogenous H_2_O_2_ to the decreased survival of the *modA2* locked ON variant compared to the *modA2* locked OFF variant. Treatment with catalase significantly reduced total ROS in the extracellular milieu (see Supplementary Fig. 1). Proteases released by neutrophils play a role in complement mediated killing of some NTHI strains^23^. To determine the contribution of exogenous proteases in the killing of NTHI strain 723, supernatants were treated with a commercially available protease inhibitor cocktail. After inhibition of proteases within the neutrophil supernatants, *modA2* locked ON was still significantly more sensitive to killing compared to *modA2* locked OFF (Fig. 5C, *P* < 0.05, unpaired t-test). From these results, we hypothesized that early during the disease course, exposure to high levels of exogenous ROS released by neutrophils, specifically H_2_O_2_, could contribute to selection for a *modA2* OFF subpopulation, as this population is less sensitive to ROS, leading to decreased killing of these bacteria and clonal expansion.

**Figure 6:**
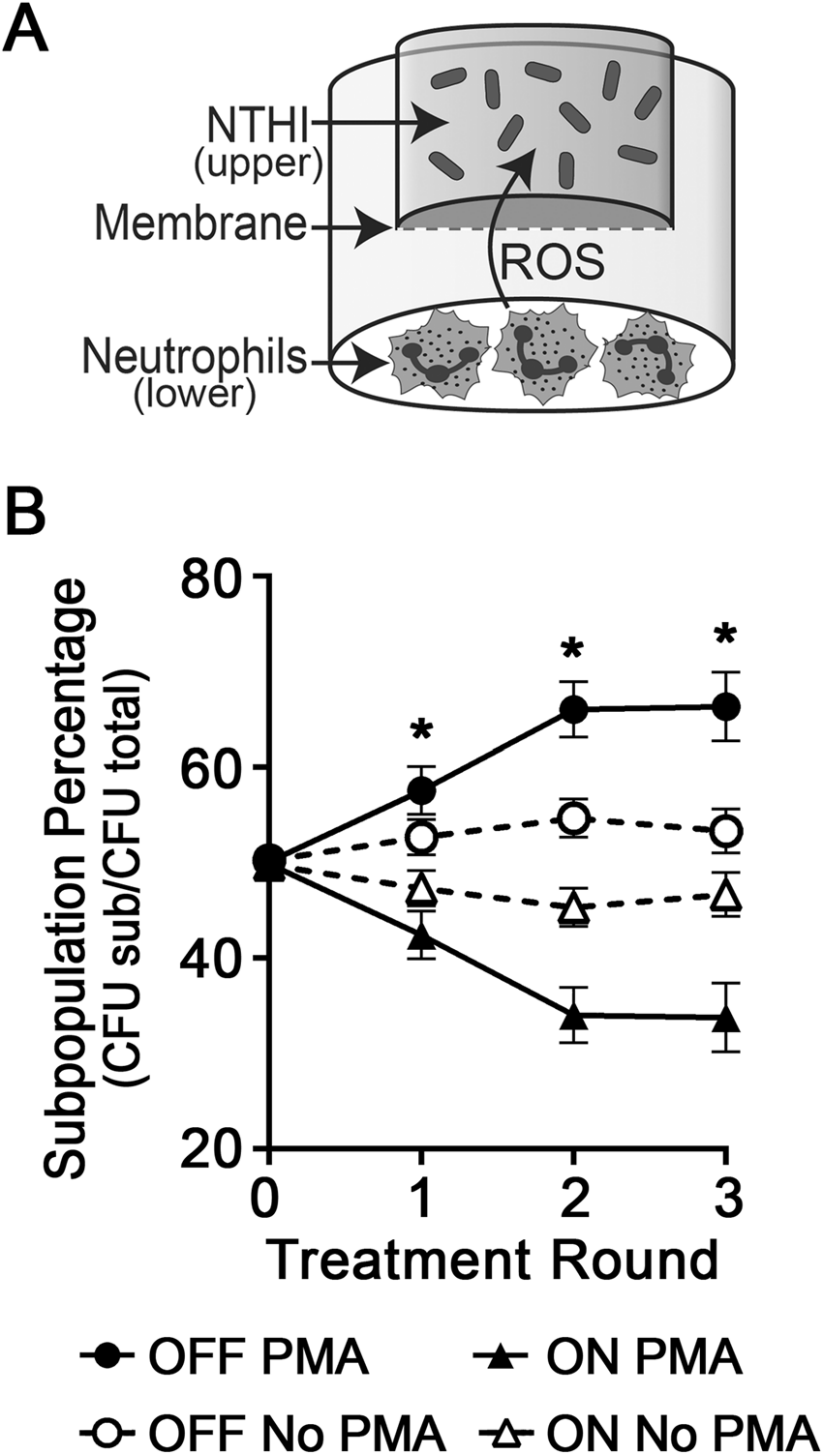
Exposure to ROS generated by human neutrophils selected for the *modA2* locked OFF subpopulation. (**A**) NTHI and activated neutrophils were physically separated by a 0.4 µM pore membrane to prevent physical contact or phagocytosis, yet permit the diffusion of ROS. Neutrophils were seeded into the lower chamber of a Transwell system and activated with 50 nM PMA for 10 minutes. NTHI were then added to the upper chamber of a Transwell and incubated for 30 min to allow exposure to ROS. (**B**) A mixed culture that contained an equal number of *modA2* locked ON:GFP and *modA2* locked OFF:mCherry was subjected to multiple rounds of exposure to neutrophil-derived ROS. The increased sensitivity of *modA2* locked ON to ROS resulted in an enrichment of the *modA2 locked* OFF subpopulation. Selection was apparent after the first round of exposure to ROS. The population percentages, *modA2* locked ON compared to *modA2* locked OFF, were significantly different after the first round, *P < 0.005, unpaired t-test. This difference remained significant after each subsequent round of exposure to ROS, *P < 0.005, multiple t-tests. These data suggest that ROS due to infiltrating immune cells will contribute to selection for the *modA2* OFF status.

To confirm that bacteria in the *modA2* OFF status were preferentially selected for over those that express ModA2 *(modA2 ON)* in the presence of neutrophil-derived ROS, we assessed mixed *modA2* ON and *modA2* OFF populations over multiple rounds of exposure to ROS. The *modA2* locked ON:GFP and *modA2* locked OFF:mCherry fluorescent reporters were again used to track each subpopulation. Human neutrophils isolated from donor blood were placed into wells of a microtiter plate and activated with PMA. Following neutrophil activation and generation of ROS, a NTHI mixed population (equal parts *modA2* locked ON:GFP and *modA2* locked OFF:mCherry) was placed in to the upper chamber of 0.4 µM pore Transwell (Fig. 6A). The Transwell system was used to physically separate the bacteria from the neutrophils, yet allow diffusion of ROS into the upper chamber of the insert. NTHI were incubated in the presence of activated neutrophils for 30 minutes and then collected from the upper chamber of the Transwell. CFU of each subpopulation were determined and bacteria were allowed to recover in the absence of neutrophils for 10 minutes. A total of three rounds of ROS exposure were performed. As was observed with H_2_O_2_ stress, the increased sensitivity of *modA2* locked ON to ROS, lead to advantageous selection of the *modA2* locked OFF subpopulation following multiple exposures to neutrophil-derived ROS (Fig. 6B, *P* < 0.005, unpaired t-tests). These results indicated that *modA2* status and subpopulation selection are likely important early in infection when NTHI experience high levels of oxidative stress due to the antimicrobial response of host immune cells.

## Discussion

The phasevarion facilitates a coordinated switch of the expression of many genes, provides a means of rapid adaptation to changes in the microenvironment, and supports evasion of host defences^7^. The ModA2 phasevarion of NTHI strain 723 regulates the expression of genes involved in iron acquisition, for which NTHI has an absolute requirement. Furthermore, anaerobic respiration and biofilm formation, which are known to be important for colonization and pathogenesis, are also regulated by the ModA2 phasevarion^8^. The involvement of the phasevarion in these critical processes highlights the global importance of this regulatory mechanism in NTHI. Many genes of the NTHI phasevarion have been identified, yet work is still needed to fully understand the role of the phasevarion in the pathogenesis of NTHI and other human pathogens. In the current study, we investigated the role of the NTHI ModA2 phasevarion in protection against oxidative stress and killing by specific innate host defences. The ability to survive oxidative stress is critical to NTHI during asymptotic persistence within the nasopharynx as well as during pathogenesis in other sites of the human respiratory tract^27, 28^.

To define the role of the ModA2 phasevarion in survival under oxidative stress, we used two variants of NTHI strain 723 in which *modA2* was either locked ON or locked OFF, and unable to phase vary. The *modA2* locked ON variant was significantly more sensitive to high levels of exogenous H_2_O_2_ and this deficiency, in turn, resulted in a selective advantage for the *modA2* locked OFF subpopulation over the *modA2* locked ON subpopulation when a mixed population was exposed to exogenous H_2_O_2_. ModA2 subpopulation selection is important, because in the context of the phasevarion, selection for an advantageous phenotype also selects for all other phenotypes associated with that subpopulation. For example, selection for a *modA2* OFF subpopulation based on fitness during oxidative stress will also alter iron acquisition and biofilm formation phenotypes^8^. Furthermore, we have shown previously that selection for a shift from *modA2* OFF to *modA2* ON within the middle ear significantly increases disease severity in a chinchilla model of OM^15^, which highlights the broad importance and impact of subpopulation selection.

In NTHI the transcriptional regulator OxyR regulates genes involved in resistance to oxidative stress, which includes the *katA* gene that encodes catalase. As catalase and OxyR have a clear role in resistance to H_2_O_2_ stress, NTHI mutants that lack OxyR or catalase were constructed to determine if either of these proteins played a role in the increased sensitivity of *modA2* ON to oxidative stress. In agreement with previous studies using other NTHI strains^18, 19^, strain 723 mutants that lacked the *oxyR* gene were significantly more sensitive to H_2_O_2_. Importantly, there was no difference in survival between the Δ*oxyR modA2* locked ON and Δ*oxyR modA2* locked OFF mutants when exposed to H_2_O_2_. Survival was also similar between the Δ*katA modA2* locked ON and Δ*katA modA2* locked OFF mutants in the presence of H_2_O_2_ stress. In fact, the *modA2* locked ON parent was just as deficient as either of the *katA* mutants. These data suggest that ModA2 expression may inhibit catalase activity, either directly via reduced transcription or indirectly through regulation of enzyme maturation and activity. Given that NTHI has multiple overlapping mechanisms to defend against oxidative stress^17^, our data suggests that mechanisms other than catalase still function in both the parent cells and the catalase mutants, but are less efficient than catalase under the conditions tested here.

In addition to enzymes that break down ROS, proteins that bind to and sequester iron are known to protect against H_2_O_2_-induced DNA damage^29^. Free iron can exacerbate oxidative stress within a cell though production of free radicals via the Fenton reaction^30^. Several NTHI genes that encode iron-binding proteins are upregulated in the presence of H_2_O_2_. These genes include *hitABC*, *hxuABC*, *tbp1/2*, and the *hfeABCD* operon, which is regulated by OxyR^19^. Intriguingly, all of these iron-binding proteins are down-regulated in NTHI strain 723 when ModA2 is expressed^8^. A decrease in the production of iron-binding proteins could potentially lead to increased free iron within *modA2* ON cells. Apart from any possible change in enzymatic activity, this increase in free iron within *modA2* ON cells could contribute to increased sensitivity of these bacteria to oxidative stress. In addition to the genes down-regulated in *modA2* ON, the primary methionine transporter *metN* is up-regulated in *modA2* ON^8^. Increased acquisition of methionine could also predispose *modA2* ON cells to be more susceptible to oxidative stress, as methionine is highly prone to oxidative damage^31, 32^. Increased methionine and free iron within the cell could severely alter cellular redox balance, and lead to a decrease in overall fitness in *modA2* ON.

Human pathogens encounter high levels of exogenous oxidative stress within the host due to the oxidative burst of granulocytes, primarily neutrophils^33^. We found that, in addition to exogenous H_2_O_2_, *modA2* locked ON was significantly more sensitive to killing by human neutrophils than *modA2* locked OFF. Neutrophils release a variety of antimicrobials in response to bacterial infection. While many of the compounds released by neutrophils are toxic to NTHI, we found that degradation of H_2_O_2_ by catalase abrogated the difference in survival of *modA2* locked ON compared to *modA2* locked OFF (Fig. 5B). Antimicrobial peptides and proteases produced by neutrophils affect NTHI survival and complement-dependent killing^23^. Treatment with a protease inhibitor cocktail did not significantly alter the increased killing of *modA2* locked ON compared to *modA2* locked OFF in the presence of antimicrobials released by neutrophils (Fig. 5C). These findings confirmed that release of H_2_O_2_ was primarily responsible for the observed differences in *modA2* subpopulation survival. Selection of the *modA2* locked OFF subpopulation within a mixed population also occurred under the pressures of neutrophil-derived ROS. Complete (100%) selection for a single subpopulation was not expected to occur with these competition experiments due to both the random nature of phase variation as well as the technical limits of these *in vitro* assays. During infection, NTHI will encounter sustained, localized oxidative stress due the continuous release of ROS from infiltrating neutrophils in the host. In our experiments, NTHI were exposed to lethal concentrations of oxidative stress for a short time and then allowed to briefly recover in the absence of stress. This recovery period was required to maintain ample viable bacterial for enumeration and subsequent rounds of exposure, but likely contributed to the observed limit in overall subpopulation selection of around 70%. Selection for specific *modA2* subpopulations can have significant effects on NTHI pathogenesis and disease severity^15^. Intriguingly, we have reported previously that the *modA2* ON subpopulation is preferentially selected for within the chinchilla middle ear during experimental OM^8^. While it is not yet fully known when or where during the course of disease the selection for a particular subpopulation occurs, we predict that the pressures of oxidative stress would have the greatest impact in the oxygen rich environment of the nasopharynx or early in infection when the innate immune defences are greatest^33^. As the middle ear fills with fluid, oxygen tension decreases and the pH becomes more alkaline^34, 35^, as a result, the selective pressures of oxidative stress will likely decrease and other pressures such as the need for nutrient acquisition or biofilm formation, will become more important. Therefore, during the course of colonization and development of disease, NTHI will encounter conditions and microenvironments that lead to a dynamic selection and counter-selection of different sub-populations of cells influenced by the prevailing environmental conditions. Under conditions of high oxidative stress/oxygen concentrations encountered in the nasopharynx, it appears that *modA2* OFF will be selected for; under the conditions encountered in the middle ear during OM, *modA2* ON will be selected for.

Several human adapted pathogens possess a phasevarion mechanism of epigenetic regulation, and all will encounter some form of oxidative stress. In *H. pylori*, loss of the phase variable type II methyltransferase M.PhyAIV results in significant down regulation of the gene that encodes catalase^12^. In *Neisseria menigitidis*, phase variation of the DNA methyltransferase ModD also alters catalase expression and resistance to oxidative stress^14^. We show here that phase variation of the ModA2 DNA Methyltransferase altered NTHI resistance to H_2_O_2_ and neutrophil derived ROS. The widespread regulation for resistance to oxidative stress by the phasevarion mechanism underscores the importance of this regulation among mucosa-associated human pathogens.

As we continue to discover new, important roles for the phasevarion in host adaptation, the importance of understanding the complexities of these systems will continue to grow. Future work should focus on determination of yet unidentified genes and phenotypes impacted by phasevarion-mediated regulation in NTHI and other human pathogens. Future studies to identify when and how microenvironmental pressures select for a particular *modA* status or subpopulation are also of great interest. Greater knowledge of how multiple diverse pressures influence subpopulation selection are necessary to fully harness the combinatorial power of the human immune system along with current and future antimicrobials to best develop treatment or prevention strategies for the diseases caused by these pathogens.

## Methods

### Bacteria and culture media used

NTHI strain 723 was received from the Finnish Otitis Media study group^36^. NTHI subpopulations that were unable to undergo phase variation (locked variants) were constructed by replacement of the wild type *modA2* AGCC repeat tract with either 1 repeat (locked ON) or 0 repeats (locked OFF). Briefly, the *modA2* gene from wild type NTHI strain 723 was amplified by PCR using primers mod-OE F (GACGACGACAAGATGAAGACAGACATTCAAACCG) and mod-OE R (GAGGAGAAGCCCGGTTATTCGCCATCTTTTTTCTCCG). The amplification product was cloned into pET46 Ek/LIC according to manufacturer’s instructions to generate pET46::modA2-16reps. Inverse PCR was carried out on pET46::modA2-16reps to generate pET46::modA2-1rep (ON) and pET46::modA2-0rep (OFF) using primers, Mod-inv-R (GTTATTGCGTTTAAAATAAATTTCTTCGCCTTC) and Mod-inv-1rep (AGCCAATTATACACTAAATTAACCCGAAAAAGACAAGAAATC), or Mod-inv-R and Mod-inv-0rep (AATTATACACTAAATTAACCCGAAAAAGACAAGAAATC), respectively. The resultant PCR products were 5′ phosphorylated with T4 polynucleotide kinase, and ligated with T4 DNA ligase (New England Biolabs, Ipswich, MA). The resultant plasmids were transformed into *E. coli* DH5alpha via heat-shock and screened with primers Him1 and Him3^8^ for the correct product size (1rep = 137 bp; 0rep = 133 bp) and then sequenced at the Griffith University DNA Sequencing Facility (GUDSF; Brisbane, Australia). The correct vectors were linearized with HincII and used to transform NTHI strain 723 via the M-IV method^37^. Following 24 hours of growth, all cells were harvested, diluted to 10^3^ CFU/mL, and plated. Transformant colonies were screened for low molecular weight PCR products of the correct size, using GoTaq (Promega) and the Him1 and Him3 primers. Variants were confirmed by DNA sequencing (GUDSF; Brisbane, Australia)

Chromosomal knock out mutations for *katA* and *oxyR* were made by allelic exchange and replacement with a kanamycin resistance cassette, as described previously^38^. Mutants were screened for kanamycin resistance and sequenced to verify allelic exchange. NTHI were routinely cultured in brain heart infusion broth [supplemented with hemin (2 µg/ml) and NAD (2 µg/ml)] (sBHI) or on chocolate agar and grown at 37 °C with 5% CO_2_ without shaking. Growth rates in sBHI broth were similar amongst all parent and mutant cultures.

Constitutive reporters were generated using the previously described plasmids pMDC-P1 and pKM1.1 (Table 1). *modA2* locked ON and *modA2* locked OFF were each transformed via electroporation with pMDC-P1 or pKM1.1, and screened for antibiotic resistance. Fluorescent reporting of each variant was confirmed on a FluorChem M (ProteinSimple, San Jose, California).

**Table 1:** List of bacteria and plasmid used in this study.

Bacteria		
NTHI strain 723	Otitis media clinical isolate	36
*modA2* locked ON	NTHI strain 723 with 1 repeat in *modA2*, unable to phase vary	This Study
*modA2* locked OFF	NTHI strain 723 with 0 repeats in *modA2*, unable to phase vary	This Study
*modA2* locked ON:gfp	*modA2* locked ON with pMDC-1	This Study
*modA2* locked OFF:mCherry	*modA2* locked OFF with pKM1.1	This Study
Δ*oxyR modA2* locked ON	*modA2* locked ON that lacks *oxyR*	This Study
Δ*oxyR modA2* locked OFF	*modA2* locked OFF that lacks *oxyR*	This Study
Δ*katA modA2* locked ON	*modA2* locked ON that lacks *katA*	This Study
Δ*katA modA2* locked OFF	*modA2* locked OFF that lacks *katA*	This Study
**Plasmids**		
pMDC-P1	*ompP5* promoter driving expression of *gfpmut3*	39
pKM1.1	*ompP2* promoter driving expression of mCherry	40
pET46 Ek/LIC	LIC-compatible derivative of pET-46b(+)	EMD Millipore
pET46::modA2-16reps	pET46 Ek/LIC containing the wild type *modA2* (16 reps) of NTHI strain 723	This Study

### *In vitro* resistance to hydrogen peroxide

Resistance to hydrogen peroxide was tested by a modification of the method described by Harrison *et al*.^17^. Briefly, NTHI were grown statically in sBHI to an absorbance of 0.45 at 490 nm. Hydrogen peroxide was added to a final concentration of 1 mM and the cultures were incubated for 10 minutes at 37 °C, then serially diluted and plated to enumerate viable bacteria. Percent survival is reported as NTHI CFU of treated cells divided by NTHI CFU of untreated cells.

### *In vitro* selection of *modA2* subpopulations due to hydrogen peroxide

The *modA2* locked ON:GFP and *modA2* locked OFF:mCherry reporters were suspended individually in sBHI and grown to an absorbance of 0.45 at 490 nm. The cultures were mixed 1:1 and 1 mM H_2_O_2_ was added to a final concentration of 1 mM, or sBHI was added a negative control. After incubation for 10 min at 37 °C with 5% CO_2_, a small aliquot was collected to be serial diluted and plated on chocolate agar. The remaining bacteria were centrifuged for 10 min at 2,683 x g, suspended in fresh sBHI, and incubated for 10 min at 37 °C in the absence of exogenous H_2_O_2_. The addition of H_2_O_2_ and subsequent steps were repeated for a total of 6 rounds. Colonies were visualized on a FluorChem M (ProteinSimple, San Jose, California) and CFU for each subpopulation were determined based on fluorescence. Subpopulation percentages were calculated as CFU of each subpopulation divided by CFU of the whole population.

### Resistance to neutrophil mediated killing

Human neutrophils were isolated from whole blood with the EasySep™ Direct Human Neutrophil Isolation Kit (Stemcell Technologies, Vancouver, BC). Neutrophils were seeded into 24-well non-tissue culture treated plates at 10^6^ neutrophils per well in 1 mL Dulbecco’s phosphate-buffered saline (DPBS). As indicated, neutrophils were pre-treated with 10 µM cytochalasin D for 10 minutes at 37 °C. Production of reactive oxygen species (ROS) was induced by addition of 50 nM phorbol 12-myristate 13-acetate (PMA) for 10 min at 37 °C. The release of ROS was verified by luminol-based detection. NTHI were grown to mid-log, added to the activated neutrophils at 10^4^ CFU NTHI per well and incubated at 37 °C for 30 minutes. Viable bacteria were recovered by addition of 100 uL 10x TrypLE (Thermo Fisher Scientific, Waltham, Massachusetts) with vigorous pipetting to release adherent bacteria and neutrophils, then serially diluted and plated to enumerate viable bacteria (CFU/well). Percent survival was calculated as CFU recovered divided by CFU of NTHI incubated in DPBS in the absence of neutrophils.

To determine which components released by neutrophils were responsible for the observed differences in NTHI killing, human neutrophils were isolated and activated as described above. Cell-free supernatants were collected 20 minutes after PMA activation of neutrophils. Catalase (1000 U/mL, MP Biomedicals, Santa Ana, California), or Protease Inhibitor (1x, Pierce, Rockford, Illinois) were added to the neutrophil-free supernatants and incubated for an additional 5 minutes at 37 °C. Supernatants without the addition of any inhibitors were incubated as is for 5 minutes at 37 °C as a control. One thousand CFU NTHI *modA2* locked ON or *modA2* locked OFF were added to 200 µL of each of the supernatants and incubated for 10 minutes at 37 °C. Bacteria were then collected and enumerated by serial dilution and plating to determine relative survival.

Methods for luminol-based determination of superoxides can be found as Supplementary Methods.

### Selection of *modA2* subpopulations in the presence of neutrophil-generated reactive oxygen species

Human neutrophils were isolated from whole blood as described above and the production of ROS was induced with 50 nM PMA for 10 minutes at 37 °C. A Transwell insert with a 6.5 mm diameter and 0.4 um pore size (Corning Incorporated, Corning, New York) was used to physically separate bacteria from the neutrophils, yet allow diffusion of ROS into the upper chamber of the Transwell insert. The *modA2* locked ON:GFP and *modA2* locked OFF:mCherry reporters were grown to mid-log, combined at a 1:1 CFU ratio and added to the upper chamber of the Transwell insert at 10^6^ total CFU in 250 µL DPBS. The complete Transwell system was then incubated at 37 °C for 30 minutes. The bacteria were collected from the upper chamber of the Transwell and a small aliquot was serial diluted and plated on chocolate agar. The residual bacteria were centrifuged for 10 min at 2,683× g, re-suspended in fresh sBHI, and incubated for 10 min at 37 °C. The bacteria were then collected by centrifugation, suspended in 250 µL of DPBS and used for addition rounds of treatment, for a total of 3 treatments. Freshly activated neutrophils in new wells were used for each subsequent round of treatment. Colonies were visualized on a FluorChem M (ProteinSimple, San Jose, California) and CFU for each subpopulation were determined based on fluorescence. Subpopulation percentages after each round of treatment were calculated as CFU of each subpopulation divided by total CFU of the whole population.

### Use of Human Blood

All blood was collected by the Nationwide Children’s Hospital Research Institute Blood Donor Service under informed consent following all institutional policies and donors remained anonymous. All research methods involving human neutrophils were approved by the Institutional Biological and Chemical Safety Committee at the Research Institute at Nationwide Children’s Hospital in accordance with local and federal guidelines and regulations.

### Statistical analysis

All statistical analyses were performed with GraphPad Prism version 6.0 (GraphPad Software, San Diego, CA). Statistical tests used and *P* values are indicated within the text and figure legends. All experiments were performed a minimum of three times on separate days in triplicate.

### Data Availability

No datasets were generated or analysed during the current study.

## Electronic supplementary material

Supplementary Information
