# Supplementary material for: The ModA2 Phasevarion of nontypeable Haemophilus influenzae Regulates Resistance to Oxidative Stress and Killing by Human Neutrophils
Source: Sci Rep. 2017 Jun 9;7:3161. doi: 10.1038/s41598-017-03552-9 (PMC5466613; doi:10.1038/s41598-017-03552-9)
Supplement: Supplementary file 1 — Supplementary Information [file 41598_2017_3552_MOESM1_ESM.pdf]

Supplementary Information

The ModA2 Phasevarion of nontypeable *Haemophilus influenzae* Regulates Resistance to Oxidative Stress and Killing by Human Neutrophils

Kenneth L. Brockman[1], M. Taylor Branstool[1], John M. Atack[2], Frank Robledo-Avila[1], Santiago Partida-Sanchez[1], Michael P. Jennings[2] and Lauren O. Bakaletz[1]

Supplementary Figures

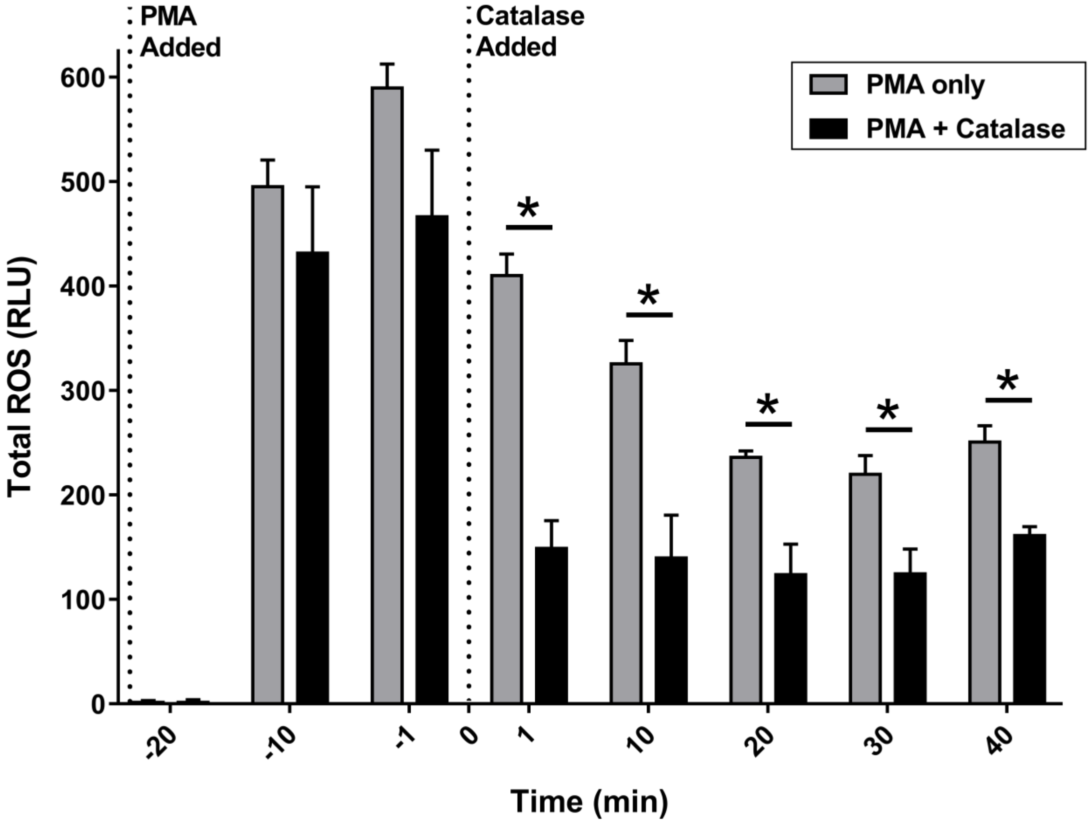

Supplementary Figure 1. Catalase treatment significantly reduces total neutrophil-derived oxidative species. Human neutrophils were activated by addition of 50 nM PMA and total ROS was measured by luminol detection. High levels of ROS were detected within 10 minutes, and peak concentrations were detected 20 minutes after addition of PMA (gray and black bars). Addition of catalase (1000 U/mL) after 20 minutes [time 0, indicated by vertical dashed line] significantly reduced total free ROS (black bars) compared to untreated neutrophils (gray bars). Total ROS was significantly decreased through 40 minutes after catalase treatment, * adjusted $P < 0.05$, unpaired t-test with Holm-Sidak multiple comparisons. These results confirm the reduction of oxidative species following catalase treatment. As catalase is specific for the degradation of H_2O_2, other oxidative species, such as superoxide, are likely responsible for the ROS detected following catalase treatment.

Supplementary Methods

Detection of total superoxide species

Human peripheral blood neutrophils were purified by negative selection (Stemcell technologies, Vancouver, BC, Canada), 10^5 neutrophils were seeded in 96 well black plates for 10 min at 37°C and incubated for 10 minutes at 37°C. Luminol (Sigma Aldrich, St. Louis, USA) was added to 100 μM and the cells were incubated for 5 minutes at 37°C and then PMA (Acros organics, Geel, Belgium) was added to a final concentration of 50 nM. Twenty minutes after stimulation with PMA, some cells were treated with 1000 U/ml of catalase (MP Biomedicals, Santa Ana, California) to remove H_2O_2. Luminiscence was measured using Synergy H1 multi-mode plate reader (Biotek, Winooski VT, USA).
